# Phylogeny and Taxonomy of the Round-Eared Sengis or Elephant-Shrews, Genus *Macroscelides* (Mammalia, Afrotheria, Macroscelidea)

**DOI:** 10.1371/journal.pone.0032410

**Published:** 2012-03-27

**Authors:** John P. Dumbacher, Galen B. Rathbun, Hanneline A. Smit, Seth J. Eiseb

**Affiliations:** 1 Department of Vertebrate Zoology and Anthropology, California Academy of Sciences, San Francisco, California, United States of America; 2 Museum of Vertebrate Zoology, University of California, Berkeley, California, United States of America; 3 Department of Botany and Zoology, University of Stellenbosch, Matieland, South Africa; 4 National Museum of Namibia, Windhoek, Namibia; Biodiversity Insitute of Ontario - University of Guelph, Canada

## Abstract

The round-eared sengis or elephant-shrews (genus *Macroscelides*) exhibit striking pelage variation throughout their ranges. Over ten taxonomic names have been proposed to describe this variation, but currently only two taxa are recognized (*M. proboscideus proboscideus* and *M. p. flavicaudatus*). Here, we review the taxonomic history of *Macroscelides*, and we use data on the geographic distribution, morphology, and mitochondrial DNA sequence to evaluate the current taxonomy. Our data support only two taxa that correspond to the currently recognized subspecies *M. p. proboscideus* and *M. p. flavicaudatus*. Mitochondrial haplotypes of these two taxa are reciprocally monophyletic with over 13% uncorrected sequence divergence between them. PCA analysis of 14 morphological characters (mostly cranial) grouped the two taxa into non-overlapping clusters, and body mass alone is a relatively reliable distinguishing character throughout much of *Macroscelides* range. Although fieldworkers were unable to find sympatric populations, the two taxa were found within 50 km of each other, and genetic analysis showed no evidence of gene flow. Based upon corroborating genetic data, morphological data, near sympatry with no evidence of gene flow, and differences in habitat use, we elevate these two forms to full species.

## Introduction

Systematists agree that the sengis or elephant-shrews represent a monophyletic family (Macroscelididae) and order (Macroscelidea) of endemic African mammals [1], with a highly distinctive evolutionary history and ecology [2]. However, the taxonomic placement of the order has proven difficult to resolve because many aspects of their morphology are unique and not similar to most other mammals [3], [4]. In the 1990s, analyses of proteins and DNA indicated that the sengis were most likely part of a relatively ancient radiation of African mammals that included several seemingly improbable clades. Now, the molecular evidence is overwhelmingly supportive of Afrotheria [5], which includes the long-recognized Paenungulata (elephants, hyraxes, and sea cows) along with the tenrecs and golden moles (order Afrosoricida), the aardvark (order Tubulidentata), and sengis (order Macroscelidea). The morphological evidence for the Afrotheria, however, continues to be weak [4], which is likely related to the approximately 105 million years that have passed since the isolation and divergence of the various afrotheres in Africa [6].

The first sengi was discovered by Western scientists at the turn of the 18^th^ C., and subsequently dozens of forms were described. The taxonomy and distribution of the various extant sengi species was reviewed and revised by Corbet and Hanks in their seminal monograph [1]. Currently, biologists recognize only 17 extant species in the order Macroscelidea [2]. The forest-dwelling giant sengis (subfamily Rhynchocyoninae with a single genus *Rhynchocyon*) includes four relatively distinct species. The soft-furred sengis (subfamily Macroscelidinae) mostly occur in arid habitats and include three genera (the monospecific *Macroscelides* and *Petrodromus*, and 11 species of *Elephantulus*).

One of the main taxonomic challenges has been resolving the sometimes minor and often cryptic phenotypic cranial and pelage differences among the many described forms. The availability of molecular techniques has changed this by clarifying taxonomic relationships that were not previously recognized, especially in the genus *Elephantulus* that contains morphologically similar species (e.g., *E. pilicaudus* from the Nama-Karoo in South Africa; [7]). However, genetic analyses have not yet been applied to many of the various forms of *Rhynchocyon*, which tend to be more easily distinguished morphologically (e.g., the recently described gray-faced sengi *Rhynchocyon udzungwensis* [8], [9]).

**Figure 1:**
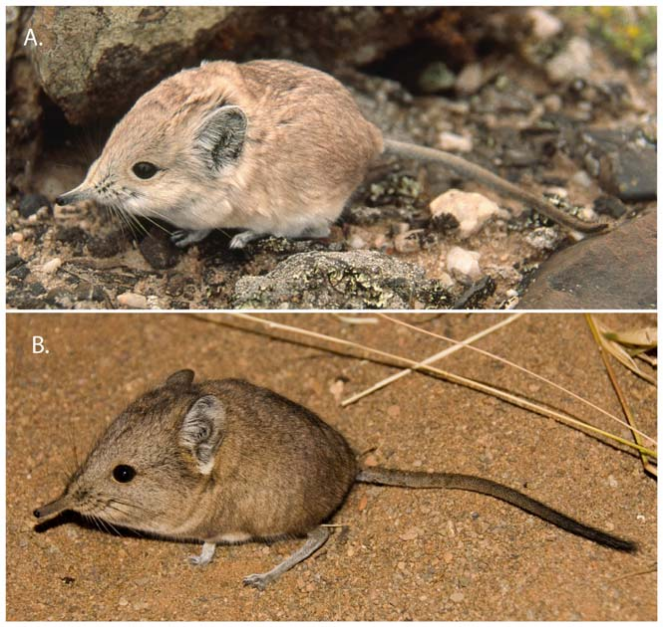
*Macroscelides* from Namibia and South Africa. A (top). *Macroscelides proboscideus flavicaudatus* captured in the Namib Desert at Wlotzkasbaken, Namibia, on 25 May 2000 (photo by GBR). B (bottom). *M. p. proboscideus* captured in the Nama-Karoo at Loxton Commonage, Northern Cape, South Africa, on 21 March 2007 (Photo courtesy of Chris and Mathilde Stuart). Note the light coloration of the animal from the Namib Desert compared to the specimen from the Nama-Karoo.

**Figure 2:**
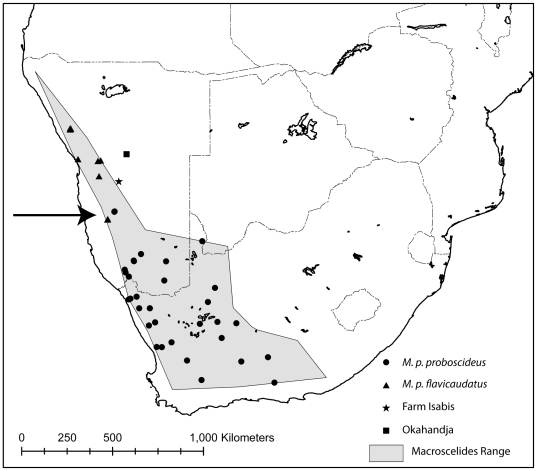
Map of southern Africa, showing the range of *Macroscelides*. Localities of specimens used in our analyses are shown (see legend and Methods). The Okahandja and Isabis localities (square and star symbols in central Namibia) of *M. p. proboscideus* are of questionable validity (see Methods), so we have not included them when calculating the distribution polygon. The arrow points to the area of potential overlap of *M. p. proboscideus* and *M. p. flavicaudatus*, between specimen localities on Gorassis (triangle) and Zwartmodder (circle) farms.

Here, we focus on members of the genus *Macroscelides* (round-eared sengis, Fig. 1), which are small (body weight 35–50 g, head and body length 104–115 mm) compared to species in the other three sengi genera [1]. They show little to no sexual dimorphism with respect to weight or external body measures [10]. Also, *Macroscelides* has a distinctively large head (due to remarkably large auditory bullae), with short and rounded ears [1], and relatively long, dense, and soft fur. Similar to other sengi genera, *Macroscelides* has large eyes, a long flexible nose, a mouse-like tail, and long spindly legs that are associated with a swift saltatorial gait [2]. *Macroscelides* is crepuscular, with an omnivorous diet [11] dominated by invertebrate prey. Like other members of the subfamily Macroscelidinae, *Macroscelides* produces small litters of highly precocial neonates and does not build or use a nest, but rather shelters in shallow burrows, among boulders, or at the bases of bushes [12]. *Macroscelides* is monogamous [13] and can occupy home ranges of up to a square kilometer [12]. *Macroscelides* is distributed from northwestern Namibia south through eastern South Africa, and occurs in extreme southwestern Botswana (Fig. 2).

*Macroscelides* is currently treated as a monotypic genus, although this masks a more complicated taxonomic history: In the 19^th^ century, four species of *Macroscelides* were described: *Sorex proboscideus* (Shaw 1800) [14]; *Macroscelides typus* Smith 1829 [15], which was renamed *Macroscelides typicus* [16]; *Rhinomys jaculus* (Lichtenstein 1831) [17]; and *Macroscelides melanotis* Ogilby 1838 [18]. Roberts [19] revised the genus, recognizing *M. proboscideus* (with nine subspecies) and *M. melanotis*. Subsequently, Lundholm [20] described a new (tenth) subspecies, *M. proboscideus flavicaudatus* from western Namibia (Table 1). Corbet and Hanks [1] only recognized a single species (*M. proboscideus*) with two subspecies, *M. p. flavicaudatus* from western Namibia and *M. p. proboscideus* from South Africa and southern Namibia, which is larger with darker pelage. Corbet and Hanks [1] suspected the variation in pelage coloration was clinal, with differences presumably being adaptations to local habitats; *M. p. flavicaudatus* being adapted to finer light-colored substrates, while *M. p. proboscideus* being adapted to darker substrates and more complex shaded habitats. Similar geographic variation in coloration has been described for *Elephantulus rufescens* in East Africa [21].

**Table 1:** Named taxa of *Macroscelides* in chronological order with contemporary descriptions of type localities [1], [19].

Taxon	Type Locality
*Sorex proboscideus* Shaw, 1800	Cape of Good Hope ( = Roodewal, Oudtshoorn Division, Western Cape)
*Macroscelides typus* A. Smith, 1829	Interior of South Africa ( = Roodewal, Oudtshoorn Division, Western Cape)
*Rhinomys jaculus* Lichtenstein, 1831	East coast South Africa
*Macroscelides melanotis* Ogilby, 1838	Damaraland, Namibia
*Macroscelides typicus* A. Smith 1838	Correct spelling of M. typus
*Macroscelides proboscideus hewitti* Roberts, 1929	Cradock, Eastern Cape, South Africa
*Macroscelides proboscideus chiversi* Roberts,1933	122 km N. Upington, Northern Cape, South Africa
*Macroscelides proboscideus langi* Roberts, 1933	Vlermuisklip, Van Rhynsdorp District, Western Cape, South Africa
*Macroscelides typicus ausensis* Roberts, 1938	32 km N. Aus, Namibia
*Macroscelides typicus brandvleinsis* Roberts, 1938	Brandvlei, Great Bushmanland, Northern Cape, South Africa
*Macroscelides typicus calvinensis* Roberts, 1938	24 km E. Calvinia, Northern Cape, South Africa
*Macroscelides typicus harei* Roberts, 1938	Brospan, mid-way between Brandvlei and Van Wyk's Blei, Great Bushmanland, Northern Cape, South Africa
*Macroscelides typicus isabellinus* Shortridge & Carter, 1938	Port Nolloth, Northern Cape, South Africa
*Macroscelides proboscideus proboscideus* Allen, 1939	Cape of Good Hope
*Macroscelides proboscideus flavicaudatus* Lundholm, 1955	9.6 km from mouth Omaruru River, Namibia

Note that *M. p. proboscideus* is a synonym of *Sorex proboscideus*, but both are listed.

While carrying out research on the behavioral ecology of sengis in Namibia [22], GBR and colleague Michael Griffin encountered several *Macroscelides* specimens from northern Namibia having darker pelage than expected, and contrasting with nearby lighter representatives of *M. p. flavicaudatus*. Additionally, other oddly colored specimens were found in the collection of the National Museum of Namibia, such as a grayish specimen (NMN 2539) from a farm (Otmarsbaum 120) near Warmbad in south central Namibia (Table S1). Concurrent with the fieldwork by GBR, HS began examining genetic variation in several southern African sengis, including *Macroscelides* [23], [24], [25]; we agreed to combine our efforts to explore the taxonomy of the genus.

With a larger and more diverse series of specimens than were available to Corbet and Hanks [1], we suspected that the size and light/dark distinction between *M. p. proboscideus* and *M. p. flavicaudatus* was not strictly clinal, and thus deserved greater attention. Based on our preliminary observations of sengis from northern Namibia that superficially resembled *M. p. proboscideus*, we hypothesized that they were either indeed closely related to *M. p. proboscideus* from South Africa (and thus represented a remarkable range extension), or they were a genetic cluster that had a unique biogeographic history (a new taxon), or that the difference in pelage color would represent adaptation to local substrates with only minor genetic differentiation (e.g., Corbet and Hanks' [1] suggestion of a cline).

To resolve these observations and hypotheses, we examined the phenotypic and mitochondrial DNA variation in the genus from the extremes of its distribution, including the morphological range of color and size differences. We included localities that provided previously named taxa, and the area where hybrid forms or clinal variation was most likely to occur. In this paper, we present our results and revise the current taxonomy for the genus *Macroscelides*.

## Methods

### Ethics Statement

All field procedures involving live animals met the standards for the ethical and humane treatment of animals of the American Society of Mammalogists [26]. Vouchered animals were euthanized using cervical dislocation, as approved by the 2000 American Veterinary Medical Association guidelines [27]. All fieldwork was performed under Research/Collecting Permit number 1177/2007 issued by the Namibian Ministry of Environment and Tourism (NMET) to JPD, and permit number 1131/2007 to GBR. Materials were legally exported under NMET export permit number 63501 to GBR.

### Distribution

We used localities of *Macroscelides* (N = 118) gleaned from museum collections, published papers, reports, and personal communications with biologists in the field [28]. We then constructed a distribution polygon (Fig. 2) using the localized convex hull (LoCoH) method [29], [30]. After applying several different K values (number of nearest neighbor hulls), we subjectively rejected those distributions with strange fragmentation and perimeters, and chose the polygon in Fig. 2, which has a K value of 20. To reduce visual clutter, Figure 2 shows only the localities of specimens that we used in our genetic and morphological analyses (Table S1), and thus many localities are not shown and some polygon corners do not include the symbol that defines them.

The latitude and longitude (presented throughout as decimal degrees south and east for all locations) for most of the voucher specimens originally analyzed and reported by HS [25] were based on the nearest town to the recorded collection location. We have georeferenced all specimens using locations from specimen labels or museum catalogs to better estimate their collection locality (Table S1), resulting in greater accuracy in Figure 2. For specimens that we collected, we georeferenced them in the field using handheld GPS units. Specimens in museum collections that contained only farm names were given the locality of the centroid of the farm shape, with the farm shape as the error. Some localities were estimated by locating the position (town or village) in the program Google Earth 5.1, and obtaining a fix. All latitude and longitude coordinates use the map datum WGS84.

We were suspicious of the collection locality for a pair of *Macroscelides* specimens labeled as “Okahandja, Namibia” from the Ditsong National Museum of Natural History (Transvaal Museum) in Pretoria, South Africa, and collected in 1950 by Walter Hoesch. Because there is no past or present farm with this name in Namibia that we are aware of, we assume that this location refers to the major town with this name. HS amplified archival tissue from one of these specimens (TM10213) and found it to be *M. p. proboscideus*. As this would be an exceptional record for this taxon, we wanted to critically evaluate its validity. The habitat near Okahandja town is unusual for this species, it being too mesic and lacking the gravel plains that *Macroscelides* typically occupies. In addition, the town is approximately 325 km north of the nearest other *M. p. proboscideus* vouchered specimen location. Recent small mammal collectors (SJE pers. obs.; C. Coetzee and M. Griffin, pers. comm.) have not captured *Macroscelides* near Okahandja. The Ditsong museum accession numbers indicate that the specimens were accessioned out of collection order (Teresa Kearney, pers. comm.) and entered into the collection with rodents that Hoesh collected on Farm Isabis (Fig. 2), some 70 km west of Rehoboth. Farm Isabis is considerably closer to the known distribution of *Macroscelides*. Although it is possible that Hoesch mislabeled specimens that were actually collected on Farm Isabis, his list of field numbers, dates, and locations do not support this. Adding to the confusion, none of his four publications on mammals in Namibia, which include information on sengis and their distributions, make reference to the Okahandja *Macroscelides* specimens [31], [32], [33], [34]. In further trying to resolve the collection locality of these two *Macroscelides* specimens, we discovered that Hoesch lost ownership of his farm northeast of Okahandja during the Great Depression and spent the rest of his career living on other farms, tutoring farm children and collecting and selling biological specimens to museums [35]. All these circumstances suggest that his sengi specimens may have been collected nearly anywhere in central Namibia. Because of the confusion over the locality of Hoesch's Okahandja specimens, we have not included them in our geographical analyses, however their location would be a significant range extension for *M. p. proboscideus*, if the Okahandja location could be confirmed (Fig. 2).

### Specimen collection in the field

To augment specimens already available to us from museum collections, in June 2007 GBR and JPD visited Namibia to collect additional material. Our choice of trapping locations was based on the proximity of the two subspecies of *Macroscelides*, where we wanted to search for a contact zone or hybrid zone. We successfully captured sengis on Zwartmodder Farm (−24.9136°, 16.2703°) near the town of Maltahohe, and on Gorassis Farm (−24.9112°, 16.2701°) in the NamibRand Nature Reserve (Table S1 and Fig. 2). These two farms are about 50 km apart, and portions of the intervening gravel plains habitat appeared suitable for *Macroscelides*, so we trapped on Keerveeder Farm (−24.9489°, 16.0394°) and Toekoms Research Station (−25.0357°, 16.0951°). Keerveeder is located roughly 22 km southwest from Zwartmodder Farm and Toekoms is approximately 35 kilometers northeast from Gorassis Farm.

We used folding aluminum Sherman live traps (8×9×23 cm) baited with a dry mixture of rolled oats, peanut butter, and Marmite (a savory yeast spread). Traps were set in transects of varying lengths with 10–20 m trap spacing. We activated the traps in late afternoon and checked them early the next morning to avoid the heat of the day. Voucher specimens were prepared as standard museum study skins with associated cranial and postcranial material, and fresh muscle and liver were preserved in 95% ethanol in the field and frozen upon return to USA.

### Genetic analyses

Our final genetic sample included a total of 78 *Macroscelides* individuals and one outgroup taxon, of which 43 were represented by tissues taken from dried museum specimens, and 35 from fresh preserved tissue (Table S1). Sampling localities were distributed throughout the range of the genus in Namibia and South Africa (Fig. 2). We had more samples of *M. p. proboscideus* than *M. p. flavicaudatus*, in part because of the difference in the extent of their distributions and in historical collecting. DNA sequences from 56 of our specimens (Table S1) were included in a phylogeographic study of *M. p. proboscideus* ([25] GenBank accession numbers EF141697–EF141822).

Total genomic DNA was extracted from alcohol-preserved frozen tissue, using a commercial DNA extraction kit (DNeasy Tissue Kit, Qiagen, Valencia, CA, USA). DNA from museum specimens (<60 years old, hereafter called archival DNA) was extracted from traditional study skins and preferentially clipped from the lip or belly skin. In some cases, tissue remnants were taken from within the skull cavity of skulls housed in museum collections. Archival DNA extractions and PCR setup were done in a dedicated “ancient DNA” facility separated from other DNA labs and all post-PCR material [36]. Some archival samples were extracted and amplified in the laboratory of JPD at California Academy of Sciences, and some by HS in the Evolutionary Genomics laboratory of T. J. Robinson and B. van Vuuren at Stellenbosch University. Extractions followed phenol/chloroform separation, and DNA was rinsed and concentrated using gravity-assisted dialysis in centricon spin columns. Extraction and PCR controls were included in all PCR experiments to test for contamination. For archival DNA, at least two independent PCR reactions were run to confirm consistent and repeatable results. See Smit *et al.* [25] and Dumbacher et al. [37], [38] for details on the laboratory extraction procedures used.

For PCR reactions, we used manufacturer's buffer (1×) as well as final concentrations of 1 µM primers, 1.2 mM MgCl_2_, 0.25 mM dNTPs, and 0.8 units Taq polymerase in 25 µl reactions in 0.2 ml tubes. Bovine serum albumin (final concentration from 0.8 to 3 mg/ml) was routinely added to amplifications to counteract PCR inhibitors present in the archival DNA samples. Amplifications used a thermal profile involving an initial denaturation step of 3–10 min at 95°C followed by 35 cycles of 95°C for 30 s, 50°C for 30 s and 72°C for 60 s. Amplifications were completed with a final 5 to 7 min extension step at 72°C. Primers were specially designed to amplify most of the mitochondrial protein coding Cytochrome *b* (Cyt *b*) gene and a fragment of the hypervariable Control Region [CR]. Primer sequences are given in Table 2. Extracts made from fresh DNA could be amplified using longer pieces. Cyt *b* primers MPL1-MPH3 (1034 base pairs) and F14164-R15181 (a 1016 base pair segment) were used to amplify roughly the same region of Cyt *b*. Degraded DNA, as found in extracts made from museum voucher specimens, required shorter amplifications, and were primed with MPL1-MPH1 (301 bases), MPL2-MPH2 (432 bases), and MPL3-MPH3 (302 bases), in three separate amplifications [25]. CR sequences were amplified with MPN1-MPD1 (338 bases) [25]. Sequencing reactions were performed using BigDye chemistry (version 3; Applied Biosystems) and analyzed on a 3100 ABI automated sequencer. Electropherograms of the raw sequences were examined and edited with Sequencher software version 4.8 through 4.10 (Gene Codes Corporation). Consensus sequences were created for each individual, aligned with Sequencher, and exported as Nexus files for further analysis.

**Table 2:** Primers used for PCR experiments.

Primer Name	Primer Sequence	Target Gene	Reference
F14164	GAAAARYCATCGTTGTAHTTCAACTA	Cytochrome b	[61]
R15181	ACWGGTTGDCCDCCRATTCAKGT	Cytochrome b	[61]
MPL1	AATCACACCCATTACTCAAAA	Cytochrome b	[25]
MPL2	TATCTACTACGGCTCCTA	Cytochrome b	[25]
MPL3	AGACCCAGACAATTATA	Cytochrome b	[25]
MPH1	GGCTACTCCGATGTTT	Cytochrome b	[25]
MPH2	GTATAATTGTCTGGGTCT	Cytochrome b	[25]
MPH3	CTAGGATTAATAKGAARTA	Cytochrome b	[25]
MPN1	CCACCATCAGCACCCAA	Control Region	[25]
MPD1	GTATAGTTCCGGTATAGAAACCCC	Control Region	[25]

Individuals of *M. p. flavicaudatus* were found to have two distinct copies of the Cyt *b* gene. To determine which was most likely the orthologous functional mitochondrial copy, we used the following techniques: 1) we searched for stop codons or frame-shift mutations that indicate a non-functional paralog, 2) we examined chromatograms for double bases that might indicate the presence of a nuclear paralog with two different alleles in a heterozygous individual, 3) we examined rates of evolution at codon positions to check whether third positions evolved most rapidly and second positions were highly conserved, as would be expected in a functional gene under selection, 4) we examined whether one gene copy had significantly slower or faster substitution rates that may indicate a nuclear paralog [39], using a Chi-squared test for significant differences in rates, and finally 5) we attempted longer PCR reactions using the most upstream Cyt *b* primers (MPL1 and F14164) and the most distant CR primer (MPD1), to amplify the largest fragment possible, and potentially identify pseudogene copies that are often smaller or that have insertions, deletions, or other problems. Because we had no evidence of any duplication in the CR region, we expected that amplifying from Cyt *b* through CR should also yield a single amplification product. The overall length of this amplification product was expected to be approximately 1250 bases.

### Mitochondrial DNA data analyses

DNA sequences were imported into PAUP* [40], and we used jModeltest [41], [42] to determine the best fit model of evolution using the Akaike Information Criterion corrected for small samples (AICc). Site-specific rate models were compared with the best-performing model from jModeltest using AICc. Maximum likelihood (ML) searches were run in PAUP*4b10 for unix using the successive iterations technique to estimate model parameters, then estimate the likelihood tree, re-estimate parameters, and so on until the parameters and tree no longer change between iterations. To estimate the support for various nodes in the tree, we used both ML fast-addition bootstrap analyses in PAUP* and a Bayesian approach using the program MrBayes 3.1 [43] run on an x-serve cluster.

Phylogenetic relationships and reciprocally-monophyletic taxonomic status were established for *M. p. proboscideus* and *M. p. flavicaudatus*. Intra- and inter-population divergences (uncorrected p-distances) as well as the number of variable and parsimony-informative sites were calculated using PAUP* [40].

### Morphological comparisons

We used 14 morphological measurements in a principal components analysis to determine if there were morphological differences among taxa. Using dial calipers calibrated to 0.1 mm, we took the following measurements in the field from captured specimens: tail length (excluding terminal hairs, from distal tip of tail to the base of the tail held at vertical right angle to dorsal aspect of head and body) and hind foot (typically the right foot from the hind edge of heel to the distal tip of the longest claw). For existing museum specimens, we took these measurements from museum tags. The following measurements were taken from prepared skulls using handheld dial calipers calibrated to 0.1 mm (for bilateral elements, the right side was used if present, and the left side was used if the right was damaged or absent, and followed the guidelines and landmarks recommended in DeBlase and Martin [44]): greatest length of skull (from the most anterior part of the rostrum to the posterior most point on the skull), greatest zygomatic breadth, least interorbital breadth, greatest breadth of braincase, height of rostrum (taken at the suture between the premaxilla and maxilla), width of bulla, greatest alveolar length of upper toothrow (including canine and incisors), greatest breadth of palate, greatest height of skull, greatest alveolar length of mandibular tooth row, height of the mandible, and greatest length of the mandible. We excluded young individuals identified by incomplete eruption of the last molars (M2) from the maxilla. Individuals with missing measurements (usually because of incomplete data on museum tags or missing or broken skull elements) also were excluded from the analysis. We analyzed the resulting data using principle components analysis and canonical linear discriminant analysis (STATA for MacIntosh, version 10.0, StataCorp, College Station, Texas).

## Results

### Specimen collection in the field

At Zwartmodder Farm, during 172 trap nights, we captured four *M. p. proboscideus*, 12 *Elephantulus intufi*, and 41 rodents. At Gorassis Farm, we had 145 trap nights and captured six *M. p. flavicaudatus*, two *Elephantulus rupestris*, and 19 rodents. *Macroscelides* were captured on relatively flat gravel plains with some structure and cover provided by small washes and scattered fist-sized rocks and cinderblock-sized boulders. Vegetation was sparse and included widely spaced dry bunch grasses and small shrubs less than 30 cm high. The gravel plains were often separated by more sandy areas or rock outcrops and mountains, which were not occupied by *Macroscelides*, but rather the bushveld sengi (*E. intufi*) or western rock sengi (*E. rupestris*), respectively. *Macroscelides* was only trapped on one transect at Zwartmodder Farm, and here it occurred with the bushveld sengi (*E. intufi*), which is normally associated with relatively flat sandy substrates that support sparse to dense bush cover. At this Zwartmodder site, gravel and sandy substrates occurred close together and resulted in captures of both species within ca. 30 m of each other.

We found no *Macroscelides* on farms between the Zwartmodder and Gorassis sites. In 315 trap-nights on Keerveeder Farm and Toekoms Research Station, we captured no *Macroscelides*, five *Elephantulus rupestris*, and 87 rodents. Thus, we found no evidence of hybrid or sympatric populations between the closest populations of the two *Macroscelides* taxa (Fig. 2).

### Sequence analyses and phylogenetics

Sequences for Cyt *b* were obtained to complement previous work done by HS and others [25] and already on GenBank (accession numbers EF141757–EF141822). Cyt *b* proved problematic in *M. p. flavicaudatus*, and we often recovered double sequences using shorter amplifications with primers MPL1-MPH1, MPL2-MPH2, and MPL3-MPH3, and sometimes with longer reactions using MPL1-MPH3. Using fresh tissue samples and long PCR reactions, we obtained clean unambiguous sequences using primers F14164-R15181 and sometimes using MPL1-MPH3. The haplotype sequences from each primer set differed by up to 0.078 (HKY85 distance, average distance 0.073 between fragments from F14164-R15181 and MPL1-MPH3 amplifications). A preliminary gene tree clearly suggested a sequence duplication in the *M. p. flavicaudatus* lineage, and efforts were made to determine whether one represented a nuclear pseudogene. Neither copy contained stop codons or frame-shift mutations that sometimes indicate a paralogous pseudogene. Neither copy appeared to have double bases that might indicate the presence of two different alleles in a nuclear paralog. Patterns of codon usage were analyzed and showed that in both copies, third positions evolved most rapidly and second positions were highly conserved. Because nuclear DNA has slower mutation rates, copies with unusually slow evolutionary rates may indicate a nuclear paralog [39]. Although the MPL1-MPH3 copy evolved more slowly than the F14164-R15181 copy, branch lengths were not significantly shorter as to indicate a nuclear copy. We then used a PCR approach using the most upstream Cyt *b* primers (MPL1 and F14164) and the most distant CR primer (MPD1). Only F14164-MPD1 gave an amplification product. When sequenced, it corresponded with the F14164-R15181 copy, and the CR portion also corresponded perfectly with the other MPN1-MPD1-primed CR sequences. Thus, we eliminated the other copy from further analysis, and proceeded with the F14164-R15181 copy. We cannot conclude with certainty that the other copy is a nuclear pseuedogene; it is possible that this is a gene copy found in the mitochondrion.

Cyt *b* sequences obtained from *M. p. flavicaudatus* museum specimens were amplified in short segments, approximately 300–400 bases. Each individual sequence clustered with one of the two *M. p. flavicaudatus* gene copies, confirming their correct taxonomic identification. For the purpose of the phylogenetic analyses, we used only the mitochondrial coding sequence, as identified by clustering with the F14164-R15181 copy in preliminary phylogenetic analsyes, and eliminated Cyt *b* sequences clustering with the duplicate Cyt *b* copy. This created gaps in the data matrix for these archival DNA samples, but avoided the possibility of creating chimeric sequences that used portions of each gene copy.

For an outgroup to *Macroscelides*, we used the only complete mitochondrial genome available from this order. The sequences are from an unidentified species of *Elephantulus* (GenBank number AB096867.1, *Elephantulus* sp.) [45]. For Cyt *b* and CR, this genome most closely matches *Elephantulus myurus* sequences (98%–99% similar) with which is it likely conspecific.

**Figure 3:**
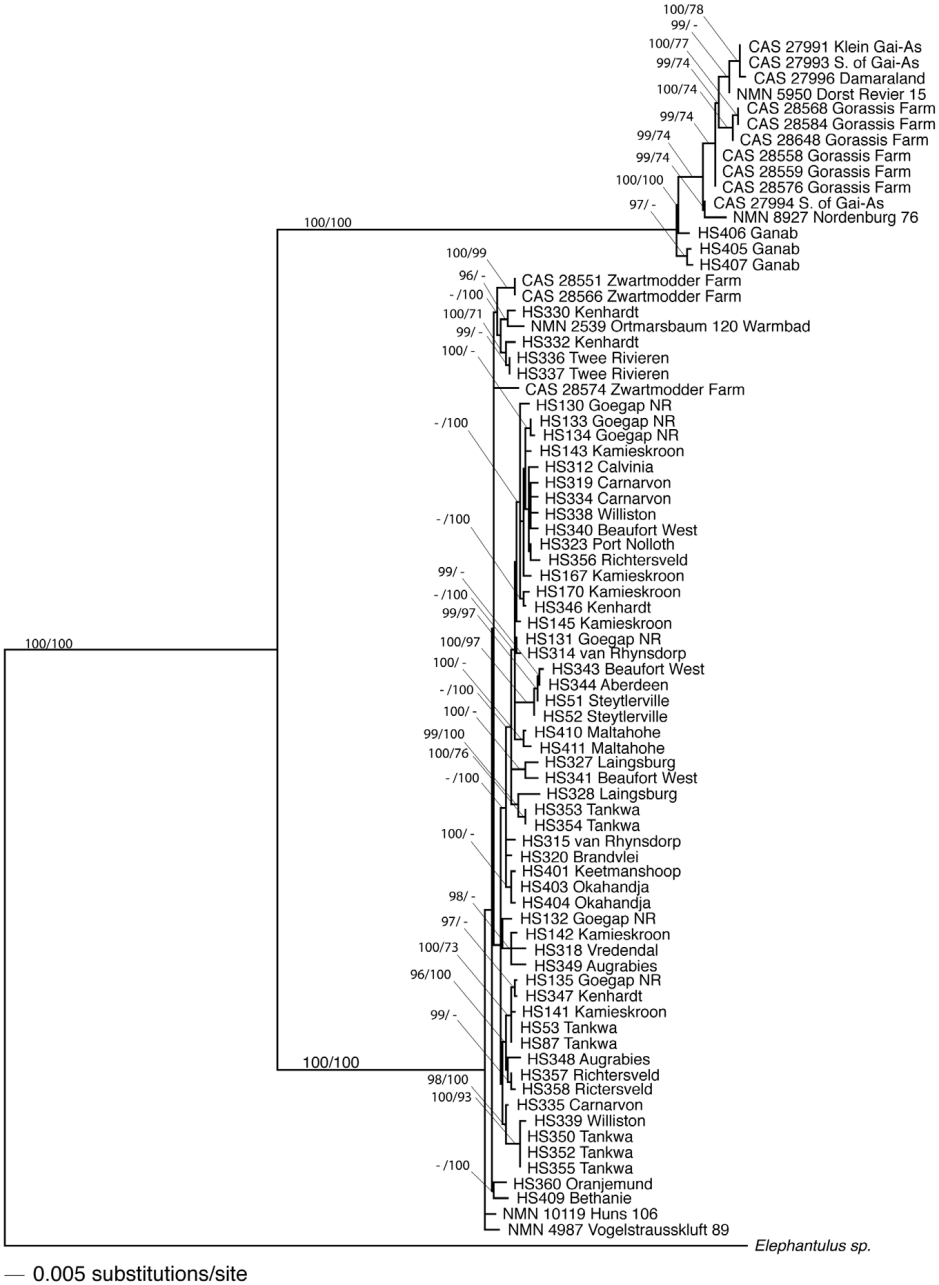
Molecular phylogeny of *Macroscelides*. Branch support is shown as Bayesian posterior probabilities (×100) followed by bootstrap values in 1000 fast-addition likelihood tree searches. Only Bayesian posteriors greater than 95 and bootstrap support greater than 70 are reported.

Using the AICc, jModeltest indicated a TIM2+I+G model of evolution with a model weight of 0.90. We additionally explored a TIM2+site specific rate model using the three codon positions of Cyt *b* and a fourth site for CR (SSR4). The site-specific rate model significantly outperformed all other models and received the model weight of 0.999. We used successive approximations of tree shape and parameters in PAUP* to find a single maximum likelihood estimate phylogeny given the TIM2+SSR4 models of evolution (Fig. 3). Parameter estimates for this search are given in Table 3. The program MrBayes was run for ten million generations using the same TIM2+SSR4 model of sequence evolution, sampling every 1000 generations. We confirmed that the potential scale reduction factor (*PSRF*) converged to one for all parameters. For analyses, the results of the first three million generations were discarded as burn-in. The resulting tree (Fig. 3) and parameter values (Table 3) were similar to the maximum likelihood analysis run by PAUP*. The Bayesian posterior probabilities were calculated from the program MrBayes are shown in Figure 3. For both gene regions, *M. p. proboscideus* and *M. p. flavicaudatus* each formed well-supported clades that were reciprocally monophyletic.

**Table 3:** Parameter estimates from phylogenetic analyses.

Parameter	Maximum likelihood	Mean (MrBayes)	95% credible interval
−Ln(likelihood)	4947.80616	5126.72	
Base frequencies:			
A	0.308362	0.304453	0.28376–0.325386
C	0.306745	0.313918	0.294273–0.333866
G	0.10933	0.10606	0.092488–0.120346
T	0.275564	0.275569	0.257436–0.294444
Rate matrix R:			
AC	1.54978	0.070678	0.051396–0.091749
AG	4.54285	0.283505	0.23189–0.338771
AT	1.54978	0.114535	0.089641–0.143007
CG	1	0.053364	0.027305–0.086909
CT	6.3631	0.387983	0.339399–0.438247
GT	1	0.089936	0.052845–0.135436
Relative Site Rates:			
Cyt b position1	0.39758	0.383505	0.300655–0.477924
Cyt b position 2	0.11616	0.114631	0.074023–0.163621
Cyt b position 3	2.59399	2.645424	2.474685–2.813977
Cont. Region	0.90202	0.868631	0.741192–1.002187

The greatest genetic distances for Cyt *b* within a *Macroscelides* subspecies were less than 2.0% uncorrected *p*-distance (see Table 4). The greatest distances within *M. p. proboscideus* were about 1.8% between individuals from among the most southern populations from Laingsburg in the Western Cape and the most northern populations from Maltahohe District and Bethanie District, in southern Namibia. Within *M. p. flavicaudatus* maximum pairwise distances were similar at 1.7%, and some of the greatest distances represent intrapopulation variation in the region near Gai-As, in the Khorixas District of the Kunene Region in northern Namibia.

**Table 4:** Summary of uncorrected p-distances within and between *M. p. proboscideus* and *M. p. flavicaudatus* based on combined data from the Cyt *b* gene and CR.

Uncorrected p-distance	*M. p. flavicaudatus*	*M. p. proboscideus*	*M. p. flavicaudatus - M. p. proboscideus*
Mean distance	0.007742989	0.009703737	0.132743109
Std Deviation	0.004407687	0.003252365	0.003004266
Max distance	0.01684039	0.01790324	0.14120071
Min distance	0	0	0.12287391

The differences between *M. p. proboscideus* and *M. p. flavicaudatus* were quite large. The minimum genetic divergence between *M. p flavicaudatus* and *M. p. proboscideus* was greater than 12%, and some haplotypes differed as much as 14% (uncorrected *p* distance). These are certainly underestimates of total divergence, as saturation and multiple substitutions are likely at these divergences. HKY85-corrected pairwise differences range from 13.7% to 16%. These differences suggest that the two taxa have been separated genetically for a significant period of time.

We examined the two copies of Cyt *b* within *M. p. flavicaudatus*. As mentioned earlier, neither gene copies contained any frame shift mutations or stop codons that could indicate a pseudogene, and codon substitution patterns matched those of coding regions under selection (third positions evolved most rapidly and second positions were most conserved). Pairwise genetic distances between the two different gene copies of Cyt *b* within *M. p. flavicaudatus* ranged from 6.2% to 7.3%, and averaged 7.0%. The duplication appears to have evolved after the split with *M. p. proboscideus*, as the distances were much less than the pairwise distance between the two subspecies, and the gene copy was never obtained from any *M. p. proboscideus* individual.

We confirmed the taxonomic identities of the two most geographically extreme specimens of *M. p. flavicaudatus* collected from northern Namibia. These included the northern most location in Namibia (Fig. 2; NMN1454, a specimen with incomplete genetic data and thus not included in the complete phylogenetic analyses) and the specimen from nearest the coast (TM10499, Table S1). For NMN1454, the Cyt *b* fragments, the Cyt *b* copy, and the CR sequences all clustered with *M. p. flavicaudatus*. From the coastal specimen (TM10499), which is the type for *M. p. flavicaudatus*, we only succeeded in sequencing CR, and this gene nested within the *M. p. flavicaudatus* clade. In addition, these two specimens both show the typical light-colored pelage (Fig. 1). This confirmed that *M. p. flavicaudatus* is the appropriate available name for this taxon and that it included all of the putative *M. p. flavicaudatus* specimens that we examined.

### Morphological principal component analysis

**Figure 4:**
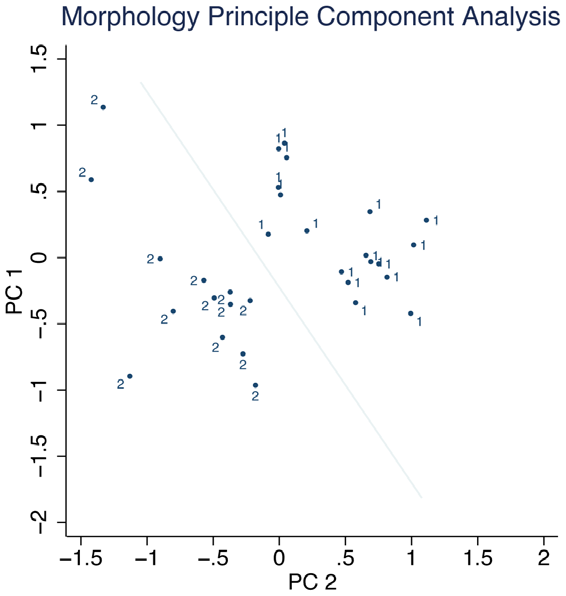
Graphical representation of morphological multivariate analysis. PC1 and PC2 are principal component axes. The numeral 1 denotes *M. p. flavicaudatus* individuals, and 2 denotes *M. p. proboscideus* individuals. The diagonal line highlights the graph region between the two putative species.

Our final complete matrix of morphological data consisted of 31 specimens for 14 characters. We performed a principal components analysis on these data, and principal components axis 1 (PC1) explained 49.65% of the variation (Table 5). All but one variable had positive loadings in PC1, and 7 variables had loadings greater than 0.3. PC2 explained an additional 15.22% of the variation. There is no overlap between the two taxa when plotted by PC1 and PC2 (Fig. 4), clearly demonstrating their separation in morphospace. The canonical linear discriminant analysis was significant (canonical correlation coefficient = 0.9735, F = 20.741, df1 = 14, df2 = 16, p<0.0001), and the primary canonical function properly classified each individual to taxon with 100% accuracy. Thus, the morphological differences between these two taxonomic groups were significant and useful for distinguishing the taxa from each other.

**Table 5:** Summary of morphological Principal Component Analysis.

Variable	PC1	PC2	PC3	PC4
Tail	−0.0878	0.4452	0.1115	0.6128
Hind Foot	0.0541	0.5612	−0.1826	0.2495
Length of skull	0.3451	−0.1925	0.0271	0.1117
Zygomatic breadth	0.3506	−0.0264	0.0463	0.0565
Interorbital width	0.0669	−0.0378	0.7123	0.1183
Breadth of braincase	0.2002	0.2428	−0.2888	0.0173
Height of rostrum	0.0618	0.3121	0.555	−0.3637
Width of bulla	0.049	−0.4636	0.1275	0.5999
Upper tooth row	0.3614	−0.0414	−0.0593	−0.0362
Breadth of palate	0.2834	0.257	0.1547	0.0258
Height of skull	0.3526	−0.0544	−0.0067	0.1398
Mandibular tooth row	0.3328	−0.0571	−0.0552	−0.0945
Height of mandible	0.346	0.0456	−0.058	−0.0534
Length of Mandible	0.3617	0.0174	−0.0495	−0.0827
PC Axis summary				
Eigenvalue	6.95	2.13	1.54	0.969
Proportion of variation explained	0.497	0.152	0.11	0.069
Cumulative proportion	0.497	0.649	0.759	0.828

Body mass was missing from many specimens, and this prohibited its inclusion in the principal component analysis. Nonetheless, we were interested in whether body mass was a useful character for determining the taxonomic status. We eliminated obvious immature individuals and performed a simple two-sample t-test with unequal variances. *M. p. flavicaudatus* (mean mass 31.5 g) was significantly lighter (p<0.01) than *M. p. proboscideus* (mean 39.0 g), although the overall ranges did overlap (*M. p. flavicaudatus*, 22–46 g, n = 18; *M. p. proboscideus*, 31–47 g, n = 13).

## Discussion

### Distribution

**Figure 5:**
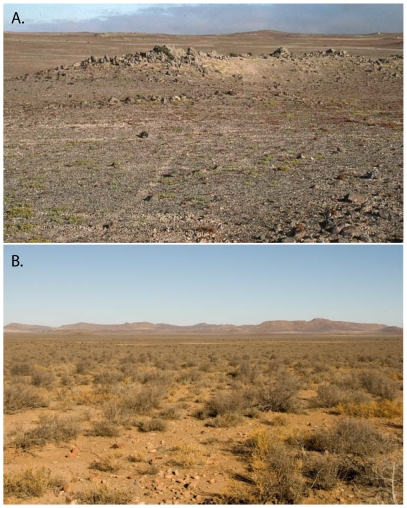
Typical *Macroscelides* gravel plain habitats in the central part of the range of each taxon. A (top). Capture site of *M. p. flavicaudatus* (see Fig. 1) in the Namib Desert near Wlotzkasbaken, Namibia (10 m elevation). Note coastal fog in distance and virtual lack of significant vegetation, except for lichens. Sengi trail is visible through center of image to rocky area in distance (Photo by GBR, 25 May 2000). B (bottom). Capture site of *M. p. proboscideus* in the Nama Karoo 40 km east of Loxton, South Africa (elevation 1364 m). Note dominance of bushes compared to the Namib Desert habitat (Photo Chris and Mathilde Stuart, 27 December 2009).

The flat to gently sloping gravel plain habitats where we captured *Macroscelides* were consistent with reports in the literature on their habitat [11], [12], [13] that suggest *Macroscelides* is a habitat specialist (see Rathbun [2] for a summary of habitat associations for different sengi species). The elevation range for the two subspecies are similar: *M. p. flavicaudatus* occurs from sea level in the central Namib to 1400 m in southern Namibia, and *M. p. proboscideus* occurs from sea level in the Succulent Karoo to 1400 m in the Nama Karoo of South Africa. However, several habitat features appear to be distinctive for each *Macroscelides* taxon. Habitats where *M. p. flavicaudatus* occurs are “desert”, which includes the Namib and Pro Namib biomes [46], [47], and are warmer than in the Karoo, largely because of the buffering effects of the ocean. Namib average yearly temperatures are 17–21°C. Namib rainfall is remarkably lower than in the Karoo, with average annual rainfall of about 15–27 mm [48]. In contrast, the gravel plains where *M. p. proboscideus* occurs are “semi-desert” in both the Succulent Karoo and the western Nama Karoo biomes [10]. The average annual temperatures in the Karoo are about 15–19°C. Snow can accumulate on the ground for a few days in some areas. The average annual rainfall is about 66–200 mm. Another distinctive difference between the two distributions, which is related to the different climatic regimes, is the dominant vegetation (Fig. 5). Surface vegetation in the Pro Namib and Namib Desert is very sparse, in some areas being dominated only by lichens that are supported by coastal fog, while in other areas sparse bunch grasses dominate in years with sufficient rainfall. Bushes of any size are highly dispersed, if present at all. In some Namib Desert habitats, where the gravel substrate is relatively coarse, *M. p. flavicaudatus* sometimes constructs and maintains distinctly straight paths between boulders [12] or rocky areas [2], which are used for shelter (Fig. 5A). In comparison, on gravel plains in the Karoo, bunch grasses and forbs can be seasonally common between scattered small (up to ca. 1 m high) bushes and bunch grasses. In addition, we found no evidence of distinctive sengi paths in *M. p. proboscideus* habitat during our 2007 fieldwork, and we are not aware that paths have been documented for this taxon elsewhere.

### Taxonomy

Our data support the taxonomic distinction of *M. p. flavicaudatus* and *M. p. proboscideus*. These two taxa form reciprocally monophyletic groups in phylogenetic analyses of mitochondrial loci. Many widely recognized full species do not achieve reciprocal monophyly [49], and so it may be a conservative criterion. Reciprocal monophyly may be misleading in small populations where gene sorting occurs rapidly despite little divergence, but this is certainly not the case for *Macroscelides*. There is significant haplotype diversity within each taxon, and the diversity is widespread.

Furthermore, mitochondrial haplotypes have diverged significantly between the two taxa with average total uncorrected pairwise sequence divergence of 13.3% (Cyt *b* only: average = 13.7%, range 12.4–14.9%; CR only: average = 12.4%, range 11.5–13.6%). This suggests that they have been evolving independently for a long time. In comparison to other groups of mammals, most species have less than 8% sequence divergence among Cyt *b* haplotypes [50], [51] and COI haplotypes [52], [53], [54], [55], with the exception of taxa thought to contain cryptic species or having multiple distinct genetic lineages.

Despite the large genetic distances, mitochondrial reciprocal monophyly, and significant multivariate morphological differences, we found no single diagnostic morphological character to distinguish the taxa. This is not unusual for members of the subfamily Macroscelidinae, as demonstrated by cryptic species in the genus *Elephantulus* [56], and is further discussed by Rathbun [2]. Initially, we hypothesized that pelage color might be diagnostic because *M. p. flavicaudatus* is generally much lighter in color than *M. p. proboscideus*. Although this is indeed the case over much of the distribution of the genus, after examining specimens at the National Museum in Windhoek, Namibia, we confirmed that there is variation in pelage darkness in both taxa, but that specimens from the Zwartmodder and Gorassis area do not show the dark/light distinction found in much of the main part of the distribution of the two taxa. Similarly, *M. p. proboscideus* generally weighs more than *M. p. flavicaudatus*, but there is variation resulting in overlapping weight ranges. Specimens from the Zwartmodder and Gorassis area largely conform to the general weight distinction, and *M. p flavicaudatus* is significantly smaller (24–29.5 g) than *M. p. proboscideus* (34–42 g) in this vicinity.

Our dataset offers no support for any of the other named *Macroscelides* taxa in the literature (Table 1). Indeed, our data support the general conclusion of Corbet and Hanks [1], that there are only two valid *Macroscelides* taxa: *M. p. proboscideus* and *M. p. flavicaudatus*. While their assessment was based on a very limited number of *M. p. flavicaudatus* specimens, our analyses include significant samples of both forms.

Although the mitochondria strongly support the two currently recognized taxa, the molecular data come entirely from a single linkage group (mitochondrial DNA), and thus should be corroborated by future studies using nuclear loci. In certain cases mitochondrial phylogenies may differ from overall species phylogenies [57], [58], so it will be important to examine nuclear gene phylogenies.

### Elevation to full species

Biological species “are groups of actually (or potentially) interbreeding natural populations which are reproductively isolated from other such groups” [59]. We searched for direct evidence of genetic mixing (hybrid forms or genetic introgression) or the absence of it (sympatric occurrence of both taxa) in the two subspecies of *M. proboscideus* by collecting in the potential overlap region between Zwartmodder Farm and Gorassis Farm in Namibia. Despite our efforts, we were unable to find any populations of *Macroscelides* between these localities. Furthermore, we genetically sampled the Zwartmodder and Gorassis populations and found no evidence of genetic mixing. We believe that the available evidence is consistent with the biological species concept, as well as the phylogenetic species concept (“the smallest diagnosable cluster of individual organisms within which there is a parental pattern of ancestry and descent” [60]) and the genetic species concept (“a group of genetically compatible interbreeding natural populations that is genetically isolated from other such groups” [50], [51]). We therefore recommend elevating the two subspecies to full species status:

*Macroscelides proboscideus* (Shaw 1800:536) with the type locality of “Cape of Good Hope” limited by Roberts (1951) to Roodeval, Oudtshoorn division, southwestern Cape Province.

*Macroscelides flavicaudatus* (*Macroscelides proboscideus flavicaudatus*) Lundholm 1955:285, with the type locality of 6 miles (9.6 km) from the mouth of the Omaruru River, South West Africa (Namibia).

In the past, the generally accepted common name for *M. proboscideus* has been the “round-eared sengi” [2]. We recommend the following common names for the new taxa. “Round-eared sengi” should now refer to the genus, which is no longer monospecific. The two new species names should include “round-eared”, so that they are clearly distinguished from sengis in other genera, plus the name of the region where each species occurs. Thus *M. proboscideus* is the “Karoo round-eared sengi” (or elephant-shrew) and *M. flavicaudatus* is the “Namib round-eared sengi” (or elephant-shrew).

### Species Traits and Conclusions

The two *Macroscelides* species diverged in mitochondrial haplotypes by an average of 13.3%, which is remarkable for two taxa that are morphologically so similar. Also, we found no evidence of *M. proboscideus* haplotypes in *M. flavicaudatus* populations, or visa versa. The two species appear to be allopatric, with about 50 km of separation in a very small area of their distribution, centered at latitude −25.0735 and longitude 16.1137 in Namibia. However, if additional collecting is done around this area, we suspect that *M. flavicaudatus* range will extend tens of kilometers to the southwest into suitable habitat in the Namib Desert, and similarly *M. proboscideus* will extend to the northeast into the Pro-Namib and Nama-Karoo. Both species occupy gravel plains, but *M. flavicaudatus* habitat in the Namib Desert and Pro-Namib tends to be much less vegetated than the Pro-Namib and Nama-Karoo gravel plains further inland, where *M. proboscideus* occurs. Although we have found no single morphological character that clearly distinguishes the two species throughout their ranges, in most areas the dorsal pelage of *M. flavicaudatus* tends to be a light buff color, whereas that of *M. proboscideus* is darker, usually being various shades of brown-gray. The body mass of the two species is also distinctive in most parts of their distribution, with the two often being separated at about 30–35 g. Interestingly, the largest *M. flavicaudatus* individuals were found far north of *M. proboscideus*, and where the two species are found nearby, they differ significantly in size. The result is that over most of the distribution of the genus, the two species are relatively easily distinguished by a combination of their location, pelage coloration, and weight.

## Supporting Information

Table S1 List of *Macroscelides proboscideus flavicaudatus* and *M. p. proboscideus* specimens used in molecular and morphological cranial analyses.
(DOCX)
