# Supplementary material for: Phylogeny and Taxonomy of the Round-Eared Sengis or Elephant-Shrews, Genus Macroscelides (Mammalia, Afrotheria, Macroscelidea)
Source: PLoS One. 2012 Mar 27;7(3):e32410. doi: 10.1371/journal.pone.0032410 (PMC3314003; doi:10.1371/journal.pone.0032410)
Supplement: Table S1 — List of Macroscelides proboscideus flavicaudatus and M. p. proboscideus specimens used in molecular and morphological cranial analyses. (DOCX) [file pone.0032410.s001.docx]

Table S1. List of *Macroscelides proboscideus flavicaudatus (=flav)* and *M. p. proboscideus (=prob)* specimens used in molecular (*) and morphological cranial (#) analyses. Provinces in South Africa (SA): NC=Northern Cape, WC=Western Cape, EC=Eastern Cape. Country abbreviations are NAM for Namibia and SA for South Africa. Museum numbers include the following codes: CAS for California Academy of Sciences, TM for Ditsong National Museum of Natural History, NMB for National Museum Bloemfontein, NMN for National Museum of Namibia, and MVZ for the UC Berkeley Museum of Vertebrate Zoology. GenBank accession numbers are provided for genetic data used in the analyses.

| Taxon | Collector No. | Museum No. | Locality | Country | Latitude south | Longitude north | GenBank cyt *b* | GenBank CR |
| --- | --- | --- | --- | --- | --- | --- | --- | --- |
| *flav^*#^* | MG4022 | CAS 27994 | Gai-As, Kunene District | Nam | 20.8000 | 14.1167 | JN560814 | JN560793 |
| *flav^#^* | MG4023 | CAS 27995 | Gai-As, Kunene District | Nam | 20.8000 | 14.1167 |  |  |
| *flav^*^* | MG4030 | CAS 27996 | Damaraland, Kunene District | Nam | 20.8367 | 14.0833 | JN560815 | JN560794 |
| *flav^#^* | GBR741 | CAS 28556 | Gorassis 99, Maltahohe District | Nam | 25.2703 | 15.9392 |  | JN560796 |
| *flav^*#^* | GBR744 | CAS 28558 | Gorassis 99, Maltahohe District | Nam | 25.2703 | 15.9392 | JN560817 | JN560797 |
| *flav^*#^* | GBR745 | CAS 28559 | Gorassis 99, Maltahohe District | Nam | 25.2703 | 15.9392 | JN560818 | JN560798 |
| *flav^*^* | TMB18 | CAS 28568 | Gorassis 99, Maltahohe District | Nam | 25.2703 | 15.9392 | JN560820 | JN560800 |
| *flav^*#^* | JPD415 | CAS 28576 | Gorassis 99, Maltahohe District | Nam | 25.2703 | 15.9392 | JN560822 | JN560802 |
| *flav^*#^* | JPD414 | CAS 28584 | Gorassis 99, Maltahohe District | Nam | 25.2703 | 15.9392 | JN560823 | JN560803 |
| *flav^#^* | GBR645 | CAS 27656 | Wlotzkasbaken, Swakopmund D. | Nam | 22.3034 | 14.4685 |  |  |
| *flav^*#^* | AB001 | CAS 27991 | Klein Gai-As, Kunene District | Nam | 20.7780 | 14.0800 | JN560812 | JN560791 |
| *flav^*^* | JPD416 | CAS 28648 | Gorassis 99, Maltahohe District | Nam | 25.2938 | 15.9261 | JN560824 | JN560804 |
| *flav^#^* |  | NMN 11330 | Gorassis 99, Maltahohe District | Nam | 25.2703 | 15.9392 |  |  |
| *flav^#^* |  | NMN 6858 | Gorassis 99, Maltahohe District | Nam | 25.2703 | 15.9392 |  |  |
| *flav^#^* |  | NMN M 3460 | Tumasberg, Namib Park | Nam | 23.15 | 15.51 |  |  |
| *flav^*^* |  | NMN M 5950 | Dorst Revier 15, Caribib District | Nam | 22.368 | 15.59 | JN560831 | JN560810 |
| *flav^#^* |  | NMN M 8925 | Nordenburg 76, Caribib District | Nam | 22.395 | 15.456 |  |  |
| *flav^#^* |  | NMN M 8926 | Nordenburg 76, Caribib District | Nam | 22.395 | 15.456 |  |  |
| *flav^*#^* |  | NMN M 8927 | Nordenburg 76, Caribib District | Nam | 22.395 | 15.456 | JN560832 | JN560811 |
| *flav^#^* |  | NMN M 8928 | Nordenburg 76, Caribib District | Nam | 22.395 | 15.456 |  |  |
| *flav^#^* |  | NMN SMM 7577 | Tumasberg, Namib Park, Swakopmund District | Nam | 23.15 | 15.51 |  |  |
| *flav^#^* |  | NMN SMM 7578* | Tumasberg, Namib Park, Swakopmund District | Nam | 23.15 | 15.51 |  |  |
| *prob^*^* | MG4017 | CAS 27993 | South of Gai-As, Kunene District | Nam | 20.8067 | 14.1167 | JN560813 | JN560792 |
| *prob^*#^* | GBR736 | CAS 28551 | Zwartmodder 101, Maltahohe District | Nam | 24.9136 | 16.2703 | JN560816 | JN560795 |
| *prob^#^* | GBR738 | CAS 28553 | Zwartmodder 101, Maltahohe District | Nam | 24.9136 | 16.2703 |  |  |
| *prob^*#^* | TMB15 | CAS 28566 | Zwartmodder 101, Maltahohe District | Nam | 24.9136 | 16.2703 | JN560819 | JN560799 |
| *prob^*#^* | JPD409 | CAS 28574 | Zwartmodder 101, Maltahohe District | Nam | 24.9136 | 16.2703 | JN560821 | JN560801 |
| *prob^#^* |  | MVZ 117060 | Garies, NC | SA | 30.55 | 17.98 |  |  |
| *prob^#^* |  | MVZ 117061 | Garies, NC | SA | 30.55 | 17.98 |  |  |
| *prob^#^* |  | MVZ 117925 | Port Nolloth, NC | SA | 29.253 | 16.984 |  |  |
| *prob^#^* |  | MVZ 117926 | Port Nolloth, NC | SA | 29.253 | 16.984 |  |  |
| *prob^*^* | HS52 | NMB12596 | Steytlerville, EC | SA | 33.3790 | 24.1903 | EF141822 | EF141756 |
| *prob^*^* | HS51 | NMB12599 | Steytlerville, EC | SA | 33.3790 | 24.1903 | EF141821 | EF141755 |
| *prob^#^* |  | NMN 10120 | Huns 106, Bethanie, Bethanie District | Nam | 27.36 | 17.24 |  |  |
| *prob^#^* |  | NMN 11227 | Zwartmodder 101, Maltahohe District | Nam | 24.9136 | 16.2703 |  |  |
| *prob^#^* |  | NMN 11228 | Zwartmodder 101, Maltahohe District | Nam | 24.9136 | 16.2703 |  |  |
| *prob^#^* |  | NMN 11230 | Zwartmodder 101, Maltahohe District | Nam | 24.9136 | 16.2703 |  |  |
| *prob^*^* |  | NMN M 2539 | Ortmarsbaum 120, Warmbad, Karasburg D. | Nam | 28.319 | 18.74 | JN560827 | JN560806 |
| *prob^#^* |  | NMN M 4683 | NamusKluft 88, Luderitz D. | Nam | 27.9204 | 16.8234 |  |  |
| *prob^*^* |  | NMN M 4987 | Vogelstrausskluft 89, Bethanien | Nam | 27.008 | 17.588 | JN560830 | JN560809 |
| *prob^*^* | HS130 | No Voucher | Goegap Reserve, Springbok, NC | SA | 29.6927 | 17.5000 | EF141782 | F141716 |
| *prob^*^* | HS131 | No Voucher | Goegap Reserve, Springbok, NC | SA | 29.6927 | 18.0267 | EF141783 | EF141717 |
| *prob^*^* | HS132 | No Voucher | Goegap Reserve, Springbok, NC | SA | 29.6927 | 18.0267 | EF141784 | EF141718 |
| *prob^*^* | HS133 | No Voucher | Goegap Reserve, Springbok, NC | SA | 29.6927 | 18.0267 | EF141785 | F141719 |
| *prob^*^* | HS134 | No Voucher | Goegap Reserve, Springbok, NC | SA | 29.6927 | 18.0267 | EF141786 | EF141720 |
| *prob^*^* | HS135 | No Voucher | Goegap Reserve, Springbok, NC | SA | 29.6927 | 18.0267 | EF141787 | EF141721 |
| *prob^*^* | HS141 | No Voucher | Paulshoek, Kamieskroon, NC | SA | 30.3948 | 18.2861 | EF141722 |  |
| *prob^*^* | HS142 | No Voucher | Paulshoek, Kamieskroon, NC | SA | 30.3948 | 18.2861 | EF141789 | EF141723 |
| *prob^*^* | HS53 | No Voucher | Tankwa National Park, NC | SA | 32.283 | 19.867 | EF141808 | EF141742 |
| *prob^*^* | HS87 | No Voucher | Tankwa National Park, NC | SA | 32.283 | 19.867 | EF141809 | EF141743 |
| *prob^*^* | Coetzee | NMN 10119 | Huns 106, Bethanie District | Nam | 27.36 | 17.24 | JN560825 | JN560805 |
| *prob^*^* | HS143 | No Voucher | Paulshoek, Kamieskroon, NC | SA | 30.3948 | 18.2861 | EF141790 | EF141724 |
| *prob^*^* | HS145 | No Voucher | Paulshoek, Kamieskroon, NC | SA | 30.3948 | 18.2861 | EF141791 | EF141725 |
| *prob^*^* | HS167 | No Voucher | Paulshoek, Kamieskroon, NC | SA | 30.3948 | 18.2861 | EF141792 | EF141726 |
| *prob^*^* | HS170 | No Voucher | Paulshoek, Kamieskroon, NC | SA | 30.3948 | 18.2861 | EF141793 | EF141727 |
| *prob^*^* | HS403 | TM10213 | Okahandja, Okahandja D. | Nam | 22.0634 | 16.8725 | EF141767 | EF141701 |
| *prob^*^* | HS404 | TM10214 | Okahandja, Okahandja D. | Nam | 22.0634 | 16.8725 |  |  |
| *flav ^*^* | HS430 | TM10499 | Omaruru River mouth, Swakopmund Dist. | Nam | 22.08 | 14.217 | EF141697 |  |
| *prob^*^* | HS330 | TM10956 | Kenhardt, NC | SA | 29.383 | 20.9 | EF141794 | EF141728 |
| *prob^*^* | HS332 | TM10958 | Rietfontein, Kenhardt, NC | SA | 28.683 | 21.25 | EF141795 | EF141729 |
| *prob^*^* | HS334 | TM12907 | Carnarvon, NC | SA | 30.37 | 21.37 | EF141800 | EF141734 |
| *prob^*^* | HS335 | TM12908 | Carnarvon, NC | SA | 30.37 | 21.37 | EF141801 | EF141735 |
| *prob^*^* | HS336 | TM16421 | Twee Rivieren, NC | SA | 26.37 | 20.62 | EF141778 | EF141712 |
| *prob^*^* | HS337 | TM16422 | Twee Rivieren, NC | SA | 26.37 | 20.62 | EF141779 | EF141713 |
| *prob^*^* | HS338 | TM27397 | Williston, NC | SA | 31.167 | 21.58 | EF141802 | EF141736 |
| *prob^*^* | HS339 | TM27398 | Williston, NC | SA | 31.167 | 21.58 | EF141803 | EF141737 |
| *flav ^*^* | HS405 | TM28898 | Ganab, Namib Park, Swakopmund D. | Nam | 23.267 | 15.5 | EF141764 | EF141698 |
| *flav ^*^* | HS406 | TM28899 | Ganab, Namib Park, Swakopmund D. | Nam | 23.267 | 15.5 | EF141765 | EF141699 |
| *flav ^*^* | HS407 | TM28906 | Ganab, Namib Park, Swakopmund D. | Nam | 23.267 | 15.5 | EF141766 | EF141700 |
| *prob^*^* | HS340 | TM29596 | Beaufort-West, WC | SA | 32.333 | 22.55 | EF141817 | EF141751 |
| *prob^*^* | HS341 | TM29597 | Beaufort-West, WC | SA | 32.333 | 22.55 | EF141818 | EF141752 |
| *prob^*^* | HS343 | TM29608 | Beaufort-West, WC | SA | 32.333 | 22.55 | EF141819 | EF141753 |
| *prob^*^* | HS344 | TM30799 | Aberdeen, EC | SA | 32.12 | 23.87 | EF141820 | EF141754 |
| *prob^*^* | HS346 | TM32460 | Kenhardt, NC | SA | 29.383 | 20.9 | EF141796 | EF141730 |
| *prob^*^* | HS347 | TM32461 | Kenhardt, NC | SA | 29.383 | 20.9 | EF141797 | EF141731 |
| *prob^*^* | HS360 | TM32634 | Oranjemund, NC | SA | 27.783 | 16.783 | EF141773 | EF141707 |
| *prob^*^* | HS409 | TM32693 | Bethanie, Bethanie District | Nam | 27.33 | 17.23 | EF141771 | EF141705 |
| *prob^*^* | HS401 | TM360 | Keetmanshoop, Keetmanshoop D. | Nam | 27.37 | 18.83 | EF141772 | F141706 |
| *prob^*^* | HS410 | TM37607 | Maltahohe District | Nam | 24.9 | 16.283 | EF141769 | EF141703 |
| *prob^*^* | HS411 | TM37625 | Maltahohe, Maltahohe District | Nam | 24.9 | 16.283 | EF141770 | EF141704 |
| *prob^*^* | HS348 | TM39303 | Augrabies, NC | SA | 29.2167 | 17.0667 | EF141780 | EF141714 |
| *prob^*^* | HS349 | TM39330 | Augrabies, NC | SA | 29.2167 | 17.0667 | EF141781 | EF141715 |
| *prob^*^* | HS350 | TM39355 | Tankwa National Park, NC | SA | 32.283 | 19.867 | EF141810 | EF141744 |
| *prob^*^* | HS352 | TM39362 | Tankwa National Park, NC | SA | 32.283 | 19.867 | EF141811 | EF141745 |
| *prob^*^* | HS353 | TM39363 | Tankwa National Park, NC | SA | 32.283 | 19.867 | EF141812 | EF141746 |
| *prob^*^* | HS354 | TM39372 | Tankwa National Park, NC | SA | 32.283 | 19.867 | EF141813 | EF141747 |
| *prob^*^* | HS355 | TM39373 | Tankwa National Park, NC | SA | 32.283 | 19.867 | EF141814 | EF141748 |
| *prob^*^* | HS356 | TM43640 | Richtersveld, NC | SA | 28.1267 | 16.9869 | EF141775 | EF141709 |
| *prob^*^* | HS357 | TM43642 | Richtersveld, NC | SA | 28.1267 | 16.9869 | EF141776 | EF141710 |
| *prob^*^* | HS358 | TM43643 | Richtersveld, NC | SA | 28.1267 | 16.9869 | EF141777 | EF141711 |
| *prob^*^* | HS312 | TM4988 | Calvinia, NC | SA | 31.383 | 19.1 | EF141804 | EF141738 |
| *prob^*^* | HS314 | TM5127 | Klawer, Van Rhynsdorp, WC | SA | 31.62 | 18.62 | EF141806 | EF141740 |
| *prob^*^* | HS315 | TM5129 | Klawer, Van Rhynsdorp, WC | SA | 31.62 | 18.62 | EF141807 | EF141741 |
| *prob^*^* | HS318 | TM7390 | Vredendal, WC | SA | 31.6129 | 18.3633 | EF141805 | EF141739 |
| *prob^*^* | HS319 | TM8257 | Brospan, Carnarvon, WC | SA | 30.4364 | 22.3058 | EF141799 | EF141733 |
| *prob^*^* | HS320 | TM8259 | Brandvlei/Williston, NC | SA | 30.467 | 20.5 | EF141798 | EF141732 |
| *prob^*^* | HS323 | TM8264 | Port Nolloth, NC | SA | 29.12 | 17.37 | EF141774 | EF141708 |
| *prob^*^* | HS327 | TM9011 | Laingsburg, WC | SA | 33.25 | 20.583 | EF141815 | EF141749 |
